# Optimizing In Vitro Efficacy Assessment of the Antisense Oligonucleotide Nusinersen in Human Cellular Models

**DOI:** 10.3390/pharmaceutics18060652

**Published:** 2026-05-26

**Authors:** Maša Sinreih Tisnikar, Alja Zottel, Katja Kristan, Tea Lanišnik Rižner

**Affiliations:** 1 Institute of Biochemistry and Molecular Genetics, Faculty of Medicine, University of Ljubljana, Vrazov trg 2, 1000 Ljubljana, Slovenia; masa.sinreih_tisnikar@sandoz.net (M.S.T.); alja.zottel@mf.uni-lj.si (A.Z.); 2 Sandoz Development Center Slovenia, Lek Pharmaceuticals d.d., Verovškova 57, 1526 Ljubljana, Slovenia

**Keywords:** nusinersen, spinal muscular atrophy, survival of motor neuron, SMN, antisense oligonucleotides, drug validation

## Abstract

**Background/Objectives:** Spinal muscular atrophy (SMA) is a rare genetic disorder caused by mutations or deletions in *SMN1*, resulting in the loss of SMN protein and severe neuromuscular consequences. Nusinersen, an antisense oligonucleotide that promotes full-length SMN2 transcript formation, has significantly improved SMA outcomes. However, standardized in vitro procedures for evaluating nusinersen efficacy remain limited. This study aimed to optimize in vitro efficacy assessment of nusinersen across two human cellular models. **Methods:** Experiments were performed using HEK293 cells and the SMA patient-derived fibroblast line GM03813. Transfection conditions were optimized for each model. In HEK293 cells, several seeding densities were evaluated for nucleofection, while in GM03813 fibroblasts, multiple transfection reagents and protocols were tested. Nusinersen activity was quantified at the transcript and protein levels, and dose–response curves were generated to determine EC_50_ values. **Results:** In HEK293 cells, a higher seeding density (1 × 10^6^ cells) yielded the most efficient nucleofection. In GM03813 fibroblasts, Lipofectamine 3000 outperformed the other transfection reagents tested. Nusinersen exhibited dose-dependent effects in both models. The EC_50_ for transcript induction in HEK293 cells was 293 nM, whereas in GM03813 fibroblasts the EC_50_ was 10 nM, demonstrating substantial model-dependent differences in response. **Conclusions:** This study establishes optimized conditions for in vitro efficacy assessment of nusinersen in HEK293 and GM03813 cellular models. These protocols provide a robust and reproducible framework for evaluating nusinersen and can be readily applied to other antisense oligonucleotides designed to correct SMN2 splicing.

## 1. Introduction

Spinal muscular atrophy (SMA) was first described in 1891 by Guido Werdnig at the University of Vienna, when he observed patients with progressive weakness in the lower extremities [1]. SMA is a genetic, autosomal-recessive disease caused by mutations or deletions in the *SMN1* gene and loss of the SMN protein [1,2,3]. Approximately 95% of the patients have a homozygous deletion of exon 7 in *SMN1* [1]. The disease occurs in 1 in 6000 to 1 in 10,000 children, and the severity of the disease depends on the number of copies of the nearly identical *SMN2* gene in individuals, as no copy of the *SMN2* gene is lethal, while five copies may be asymptomatic [1]. Based on the age and severity of the disease, SMA can be classified into different types (I, II and III), but some experts propose an expanded classification that includes additional subtypes [4]. Type I (also known as Werding–Hoffman disease) is the most severe form, with onset before six months of age and death occurring in the first two years of life and accounts for approximately 60% of all SMA cases [5]. In type II, symptoms appear before 18 months of age, and patients never acquire the ability to walk. Life expectancy is shortened, and patients continuously lose upper body strength and sometimes respiratory function. In type III (known as Kugelberg–Welander syndrome), symptoms usually appear after 18 months of age, and patients are able to stand and walk.

**Figure 1:**
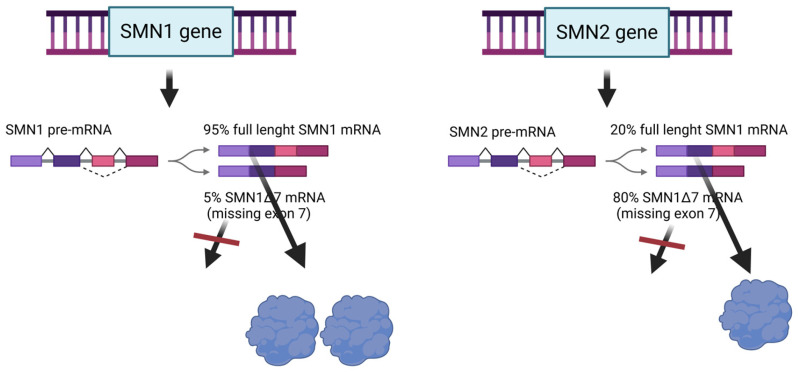
Genetic background of SMA. Created in BioRender. Sinreih, M. (2026) https://BioRender.com/g3vwhbu, accessed on 17 April 2026.

Humans have two copies of the *SMN* gene: *SMN1* (telomeric gene) and *SMN2* (centromeric gene), and the development of SMA correlates with homozygous absence of telomeric *SMN1*. *SMN1* and *SMN2* differ in 11 nucleotide substitutions: seven in intron 6, two in intron 7, one in coding exon 7, and one in non-coding exon 8. Exon 7 is efficiently included in the spliced mRNA of the *SMN1* gene; however, a silent C6T transition in *SMN2* exon 7 results in the skipping of this exon during pre-mRNA splicing [6]. As a result, *SMN2* encodes the exon 7-skipped protein isoform SMNΔ7, which is unstable, mislocalized and at best only partially functional. However, approximately 15–20% *SMN2* transcripts are correctly spliced, resulting in a functional SMN protein, but the small amount of the functional full-length protein cannot fully compensate for the loss of the *SMN1* gene [3,7,8,9]. The genetic background of the disease is schematically shown in Figure 1. Correction of exon 7 splicing has been shown to be a therapeutically effective way for introducing the functionality of the protein encoded from the *SMN2* gene [10]. A drug based on this mechanism that has provided a breakthrough in the treatment of SMA is nusinersen, a steric block antisense oligonucleotide drug [11]. It is the first drug approved for the treatment of SMA and has shown improvement in motor function in all SMA types [12,13,14]. It is an oligonucleotide consisting of 18 nucleotides that prevents *SMN2* splicing by binding to a specific sequence in the intron of exon 7 of the pre-mRNA, resulting in expression of the full-length SMN transcript [15]. Nusinersen is fully modified with 2-O’-methoxyethyl ribose, which increases resistance to nuclease degradation and improves stability and half-life, 5-methylcytidine bases, and a full phosphorothioate backbone linkage that further enhances nuclease resistance and promotes interactions with cellular proteins [16].

Despite extensive clinical and preclinical use of nusinersen, there is currently no standardized or broadly accepted in vitro framework for evaluating the efficacy of antisense oligonucleotides targeting *SMN2* splicing. Published studies frequently employ different cellular models, cell-seeding densities, and transfection strategies, often selected empirically and without systematic comparison. This methodological heterogeneity hampers reproducibility and complicates the interpretation and comparison of quantitative efficacy parameters, including EC_50_ values. Because in vitro efficacy data are commonly used for candidate selection and comparative evaluation of novel antisense oligonucleotides, the absence of optimized and clearly defined experimental conditions represents a significant methodological gap.

The aim of this study was therefore to systematically optimize and benchmark in vitro experimental conditions for assessing nusinersen activity in two widely used human cellular models: HEK293 cells and the SMA patient-derived fibroblast line GM03813. HEK293 cells were employed as a robust and reproducible heterologous system for evaluating the mechanistic effects of nusinersen on *SMN2* splicing under well-controlled transfection conditions, whereas GM03813 fibroblasts were used as a disease-relevant model to assess nusinersen activity in an endogenous SMA genetic context. By defining robust delivery conditions, identifying key experimental determinants of efficacy, and linking these parameters to transcript- and protein-level readouts, this work proposes a practical framework applicable to the evaluation of both established and emerging *SMN2*-targeting antisense oligonucleotide therapeutics.

Importantly, the novelty of this study does not lie in the identification of new biological effects of nusinersen, but in the systematic dissection of experimental variables that determine the robustness, reproducibility, and quantitative outcome of in vitro antisense oligonucleotide efficacy assays.

## 2. Materials and Methods

### 2.1. Cell Lines

In our study, the experiments were performed on two different model cell lines, HEK293 and GM03813.

HEK293 is a cell line derived from human embryonic kidney cells by the transformation and culturing of normal cells with sheared adenovirus type 5 DNA. The cell line was purchased from ECACC and arrived at passage 59 (Acc. No: 85120602, lot. 18H003, the authentication by STR profiling performed by ECACC in October 2018). Cells were grown in Eagle’s minimum essential medium with Earl’s salts (MEM, Sigma Aldrich, St. Louis, MO, USA) and non-essential amino acids (Sigma Aldrich) supplemented with 10% of fetal bovine serum. All experiments were performed below +20th passage.

GM03813 cells originate from a 3-year-old boy with type 2 spinal muscular atrophy. The cells were established by Scudiero et al. in 1986 [17]. The donor has three copies of the *SMN2* gene, and PCR analysis showed that this subject is homozygous for the deletion of exons 7 and 8 in the *SMN1* gene [18]. GM03813 cells were purchased from Coriell Institute and arrived at the 5th passage in April 2021. Cells were grown in Eagle’s minimum essential medium with Earl’s salts (MEM, Sigma Aldrich) and non-essential amino acids (Sigma Aldrich) supplemented with 15% of fetal bovine serum. All experiments were performed below +20th passage.

Both of the cell lines tested negative for Mycoplasma using the MycoAlert™ kit (Lonza, Basel, Switzerland).

### 2.2. Antisense Oligonucleotides

Two nusinersen sources (Table 1) and two negative control oligonucleotides with phosphorotioate backbone, 5-methylcytidines and methoxy ethyl base modifications (Axolabs, Kulmbach, Germany) were used (Table 2) in the experiments. Two independent batches of nusinersen were used to assess the reproducibility of antisense oligonucleotide activity under the optimized experimental conditions and to exclude potential batch-specific effects.

### 2.3. Transfection Optimization

#### 2.3.1. Electroporation of Oligonucleotides to HEK293 Cells

Nucleofection was used to transfect the HEK293 cells with the oligonucleotides. We also performed a control experiment, where no oligonucleotides were added. We plated three different concentrations of cells/well, 2 × 10^5^, 5 × 10^5^ and 1 × 10^6^, to determine the optimal number of cells per experiment. First, 1.4 mL/well of complete cell medium was pipetted into a 6-well plate, and the plate was placed in the incubator to keep the medium warm. The cells were counted, transferred to microcentrifuges and centrifuged for 5 min at 1000 rcf. Media was aspirated, and cells were suspended in 20 μL of SF Cell Line Nucleofector^®^ Solution and Supplement 1 mixture (part of SF Cell Line 4D-NucleofectorTM X Kit S, Lonza, cat. V4XC-2032). Then, 1 μL of oligonucleotides (200 μM) was added to the cells (final concentration 10 μM), and the mixture was transferred to 16-well Nucleocuvette^®^ Strips (part of SF Cell Line 4D-NucleofectorTM X Kit S, Lonza, cat. V4XC-2032), and program CM130 (transfection program for HEK293 cells) on 4D Nucleofector System (Lonza) was applied. After transfection, 80 μL of warm full medium was added to the cells, and the mixture of cells, oligonucleotides and medium was transferred to the 6-well plate with prewarmed complete cell medium (1.4 mL/well). After 72 h of incubation, cells were washed twice with PBS and proceeded to RNA or protein isolation.

#### 2.3.2. Transfection in GM03813 Cells Using Different Transfection Reagents

For the GM03813 cell line transfection, five different protocols were tested. In all cases, on the first day, 120,000 cells/well were seeded in a 12-well plate. The next day, cells were washed with 1 mL of Opti-MEM (Gibco (Waltham, MA, USA), cat. 51985026), and 700 μL of Opti-MEM was added. Afterward, cells were returned to the incubator until oligonucleotide treatment (approx. 30 min). The starting concentration of the oligonucleotides was 80 μM, and the final concentration (per well) was 200 nM.

For lipofection with Lipofectamine 3000 without the addition of p3000 reagent, two different concentrations of Lipofectamine 3000 reagent (ThermoFisher (Waltham, MA, USA), cat. 11668027) were used. Complexes with Lipofectamine and oligonucleotides (conc. 80 μM) were prepared using 1.5 or 3 µL of Lipofectamine reagent and 2 µL of oligonucleotides, each diluted in 50 µL of Opti-MEM and combined. Complexes were incubated for 10 min at room temperature and then added to the wells (100 μL/well).

When lipofection with Lipofectamine 3000 with the added p3000 reagent (a part of the Lipofectamine 3000 kit, Invitrogen (Thermo Fisher Scientific, Carlsbad, CA, USA), cat. 11668027) was performed, complexes with Lipofectamine and oligonucleotides were prepared by combining 3 µL of Lipofectamine reagent in 50 µL Opti-MEM and 2 µL of oligonucleotides (80 μM) with 2 µL of p3000 reagent in 50 µL Opti-MEM. Complexes were incubated for 10 min at room temperature and then added to the wells (100 μL/well).

For lipofection with Lipofectamine 2000 (Invitrogen, cat. L3000008), complexes with Lipofectamine and oligonucleotides were prepared by adding 5 μL of Lipofectamine 2000 reagent in 50 μL Opti-MEM to 2 μL of oligonucleotides (80 μM) in 50 μL Opti-MEM. The complexes were incubated for 5 min at room temperature and then added to the cells (100 μL/well).

For all Lipofectamine combinations, Opti-MEM medium was replaced with full culture medium after 6 h of incubation. After 72 h of incubation, cells were washed twice with PBS and proceeded to RNA or protein isolation.

When PepMute (SignaGen Laboratories (Frederick, MD, USA), cat. SL100566) was used for transfection, the day after cell seeding, cells were washed with 1 mL of PBS and 675 μL of full culture media was added 30–60 min before adding PepMute complexes, then cells were returned to the incubator. Before use, the PepMute buffer was diluted 1:4 in sterile water. Complexes were prepared by adding 2 µL of oligonucleotides (80 μM) and 2 µL of PepMute reagent to 75 µL of PepMute buffer. Complexes were incubated for 15 min at room temperature and then added to the cells (75 μL/well). The next day, the medium was aspirated, and 1 mL of fresh full MEM medium was added. After 48 h of incubation, cells were washed twice with PBS and proceeded for protein or RNA isolation. (Please see Supplementary Tables S1–S4 for summaries of the different protocols).

### 2.4. RNA Isolation and qPCR

The total RNA was isolated and purified using Nucleospin RNA isolation kits (cat. 740955.5, Macherey-Nagel GmbH & Co. KG, Düren, Germany), according to the manufacturer’s instructions. The concentration of RNA was determined using NanoDrop (ThermoFisher). The RNA quality and purity were determined by measuring A260/A280 and A260/A230 using NanoDrop (ThermoFisher) and by Agilent 2100 Bioanalyzer with the RNA 600 Nanokit (Agilent Technologies Inc., Santa Clara, CA, USA). Samples of total RNA (0.5 µg) were reverse transcribed into cDNA (in 10 µL volume) using a SuperScript^®^ VILO™ cDNA Synthesis kit (cat. 11754050, Invitrogen, Thermo Fisher Scientific, Carlsbad, CA, USA), according to the manufacturer’s instructions. For qPCR, all samples were run in technical triplicates, using 0.25 μl cDNA/well, and the reactions were performed in Applied Biosystems^®^ MicroAmp^®^ Optical 384-well plates (Thermo Fisher Scientific, Waltham, MA, USA), in a reaction volume of 5.0 μL, using TaqMan^®^ Fast Advanced Master Mix (cat. number 4444963) and universal thermocycling parameters recommended by Applied Biosystems (1 cycle of 20 s at 50 °C, 1 cycle of 20 s at 95 °C, 45 cycles of 1 s at 95 °C and 20 s at 60 °C). Quantification was accomplished with the Applied Biosystems^®^ ViiA™ 7 Real-Time PCR System (Thermo Fisher Scientific, Waltham, MA, USA). The assays are listed in Table 3 and Table 4 and were published by Naryshkin et al. [21].

The PCR amplification efficiency was determined from the slope of the log-linear portion of the calibration curve for each gene investigated, and this was accounted for in the further calculations. For gene expression analysis, the normalization factor for each sample was calculated based on the geometric mean of the reference genes (*HPRT1*, *POLR2A*, *GAPDH*) [20,22,23]. Gene expression for each sample was calculated from the crossing-point value (Cq) as *E*^−Cq^, divided by the normalization factor. The expression was determined in three biological replicates. MIQE guidelines were considered in the performance and interpretation of the qPCR reactions [24]. The list of assays is shown in Table 3 and Table 4.

### 2.5. Protein Isolation and Western Blot

The concentration of proteins isolated from the cells was measured according to the manufacturer’s instructions using ROTI Nanoquant (Carl Roth K880.1, Karlsruhe, Germany) reagent and Standard Set BSA (Biorad 500-0207, Hercules, CA, USA). Protein aliquots of samples and protein standard ladder (5 μL, Nippon Genetics MWP04, Tokyo, Japan) were separated by SDS PAGE on Mini-PROTEAN Precast 10% gels (Bio-Rad 4561035, batch: 64394032) at 180 V for 1 h–1 h 15 min. The proteins were transferred to PVDF membranes (Millipore IEVH85R (Burlington, MA, USA), lot. ROPB40697) at 108 V for 1 h 15 min and blocked with 5% nonfat milk (Merck (Darmstadt, Germany), cat. 70166) for 2 h to avoid non-specific binding. The membranes were incubated overnight with the primary anti-SMN antibodies at 4 °C (Table 5). The next day, the membranes were incubated with the secondary antibodies (peroxidase-conjugated goat anti-mouse IgG + IgM [H + L], 1:10,000; Jackson ImmunoResearch Laboratories Inc., West Grove, PA, USA, cat. 115-035-062) for 2 h. Supersignal West Pico or Supersignal West Femto Chemiluminescence Substrates (Thermo Fischer Scientific; Rockford, IL, USA) were used for the detection of signals, according to the manufacturer’s instructions, using a Fujifilm LAS4000 digital imaging system (Fujifilm; Tokyo, Japan). HepG2 cell lysate (prepared in our lab), SMN human recombinant protein (Origene (Rockville, MD, USA), cat. TP321367), and SMN human over-expression lysate from HEK293T cells (Origene, cat. LY424781) were used as positive controls, and plasma proteins (Sigma, cat. P9523, lot. SLBX 8880) were used as a negative control. SMN Δ7 recombinant protein (Abnova (Taipei City, Taiwan), cat. H00006607-P01) was an additional control.

For normalization, anti-α-tubulin antibodies (Abcam (Cambridge, UK), cat. Ab52866, lot GR3241238-9) were used. As described above, the membranes were incubated overnight with the primary antibodies at 4 °C. The next day, the membranes were incubated with the secondary antibodies (peroxidase-conjugated goat anti-rabbit IgG + IgM [H + L], 1:10,000; Jackson ImmunoResearch Laboratories Inc., West Grove, PA, USA, cat. 111-035-045) for 2 h.

Four different antibodies against SMN were examined, as described in Table 5.

### 2.6. Statistical Analysis

Statistical analysis was performed using GraphPad Prism version 10.1.2 for Windows, GraphPad Software, USA. Normal distribution of gene expression data (four values) was determined by Shapiro–Wilk test. For statistical analysis, 1-way Anova with Dunnett’s multiple comparison test was used for data with normal distribution and Kruskal–Wallis with Dunn’s multiple comparison test for data without normal distribution. A *p*-value below 0.05 was considered statistically significant.

## 3. Results

The experimental strategy was designed to address the variability and lack of standardization of published in vitro assays for evaluating antisense oligonucleotide efficacy in spinal muscular atrophy (SMA) models. Because transfection efficiency, cytotoxicity, and productive intracellular delivery are highly cell-type dependent, key experimental parameters were optimized separately for each cellular model used in this study.

HEK293 cells were employed as a robust and reproducible heterologous system to evaluate the mechanistic effects of nusinersen on *SMN2* splicing under tightly controlled transfection conditions. As nucleofection in HEK293 cells is associated with substantial cytotoxicity, optimization in this model focused on cell seeding density to ensure comparable post-transfection confluency and reproducible functional readouts.

In contrast, GM03813 patient-derived fibroblasts, which exhibit slower proliferation rates and increased sensitivity to electroporation, were used as a disease-relevant cellular model. Optimization in this system, therefore, focused on identifying lipid-based delivery strategies that balance transfection efficiency and tolerability, while maintaining constant cell-seeding density to reduce experimental variability.

Because intracellular delivery efficiency is inherently influenced by experimental conditions, differences observed between tested conditions are interpreted as reflecting condition-dependent effects on productive oligonucleotide delivery and downstream biological response. In this context, delivery efficiency was evaluated functionally through *SMN2* splicing correction, SMN protein expression, and assessments of cell morphology and viability. The results, therefore, illustrate how parameters such as cell seeding density and transfection strategy modulate functionally relevant SMN readouts under controlled in vitro conditions.

### 3.1. Cell Line Characterization

Because GM03813 cells lack functional *SMN1* and rely exclusively on *SMN2* for SMN protein production, two *SMN2*-derived transcripts were quantified throughout this study: the exon 7-inclusive, full-length SMN2 transcript (FL-SMN) and the exon 7-skipped transcript (SMN2Δ7). For clarity, transcript labels shown as “SMN1” and “SMN2” correspond to FL-SMN (exon 7-inclusive) and SMNΔ7 (exon 7-skipped) transcripts, respectively, as defined above.

**Figure 2:**
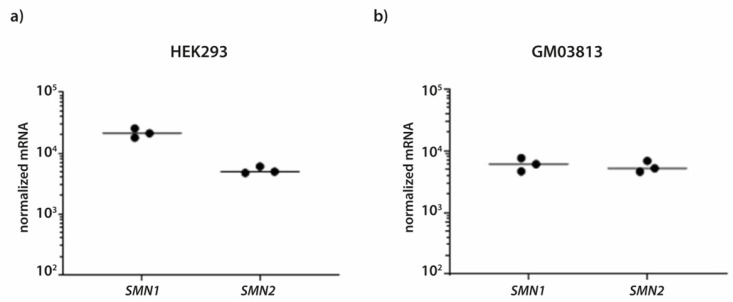
Endogenous levels of *SMN1* and *SMN2* determined by qPCR. The figure presents the endogenous levels of *SMN1* and *SMN2* in HEK293 (**a**) and GM03813 (**b**).

First, the endogenous levels of *SMN1* and *SMN2* in cell lines were determined (Figure 2). While both cell lines express *SMN* genes, the results show that the levels of *SMN1* and *SMN2* in GM03813 are comparable, while in HEK293, the level of *SMN1* is approximately 3-fold higher compared to the level of *SMN2*.

### 3.2. The Effect of Nusinersen on SMN1 and SM2 Expression in HEK293 Cells

For the transfection of oligonucleotides in HEK293, nucleofection was applied. We transfected the cells at the 10 μM final concentration of oligonucleotides at three different cell seeding concentrations: 2 × 10^5^/well, 5 × 10^5^/well and 1 × 10^6^/well. Two negative controls were used, sequence 4 and sequence 5. For comparison, cells without any oligonucleotide were also used as a control. Cells were harvested 72 h after nucleofection. The morphology of cells was observed in 24 h intervals (Supplementary Figures S1 and S2). The figures indicate that the suitable confluence is achieved with the highest seeding number (1 × 10^6^ cells/well).

**Figure 3:**
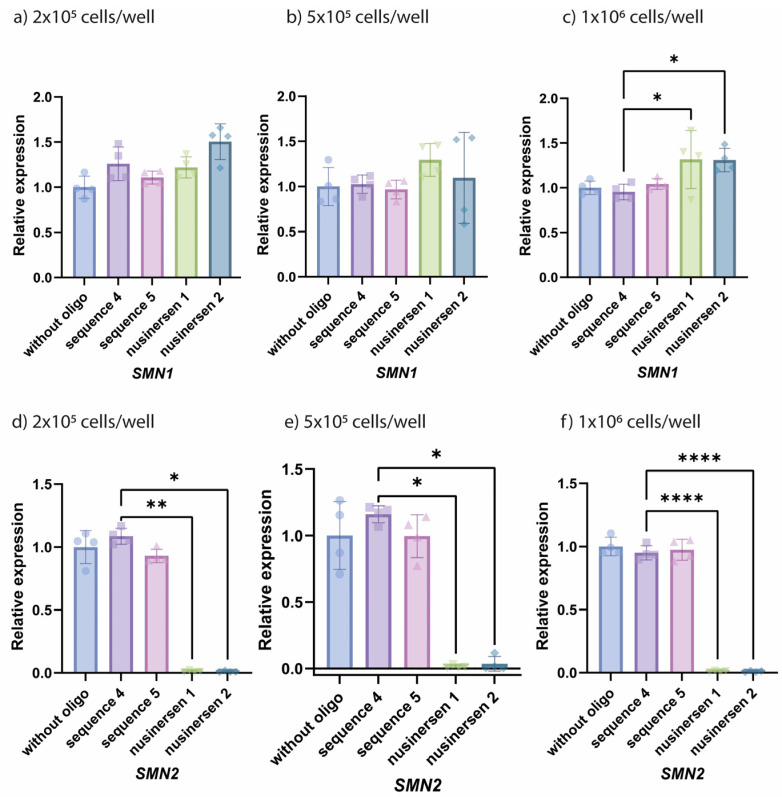
The effect of the nusinersen (final concentration 10 μM) and control oligonucleotides on *SMN1* and *SMN2* gene expression in HEK293. Gene expression was normalized to control (no oligonucleotides added). The mean of each column was compared with the mean of the control column (Sequence 4). * *p*  <  0.05, ** *p*  <  0.01, **** *p*  <  0.0001. Results of at least three independent experiments in two replicates are shown as mean ± SD. For statistical analysis, 1-way Anova was used for data with normal distribution and Kruskal–Wallis test for data without normal distribution. Results were compared to sequence 4.

The effect of nusinersen on *SMN1* (full-length transcript) and *SMN2* (truncated transcript) expression was evaluated by qPCR. The results show that at a seeding density of 2 × 10^5^/well and 5 × 10^5^/well, no statistical significance was observed for *SMN1* (Figure 3a–c). At the highest seeding concentration, 1 × 10^6^, the expression of *SMN1* in the presence of both lots of nusinersen was significantly higher compared to sequence 4. In contrast, the expression of *SMN2* decreased significantly, by 97–99%, and there were no differences between the cell-seeding numbers (Figure 3d–f). As expected, no differences in biological activity were observed between the two nusinersen batches. The results indicate that the higher cell-seeding concentration is preferred for optimal results.

### 3.3. The Effect of Nusinersen on SMN1 and SMN2 Gene Expression in GM03813 Cells

Next, we determined the expression of *SMN1* and *SMN2* in GM03813 after treatment with both nusinersen 1 and nusinersen 2. Sequence 4, sequence 5 and no addition of oligonucleotides were used as controls. For transfection, we tested several protocols/reagents: Lipofectamine 3000 with a higher concentration of Lipofectamine (3 μL/well) and no addition of p3000 reagent, Lipofectamine 3000 with a lower concentration of Lipofectamine (1.5 μL/well) and no addition of p3000 reagent, Lipofectamine 3000 with a higher concentration of Lipofectamine (3 μL/well) and addition of p3000, and Lipofectamine 2000 and PepMute. The morphology of cells was monitored in 24 h intervals (Supplementary Figures S3–S5). The results show that Lipofectamine 3000 and PepMute do not change the morphology of the cells, while Lipofectamine 2000 in the presence of oligonucleotides is highly cytotoxic to cells. Cytotoxicity is related to the combination of Lipofecatmine 2000 and oligonucleotide and is not oligonucleotide-specific.

**Figure 4:**
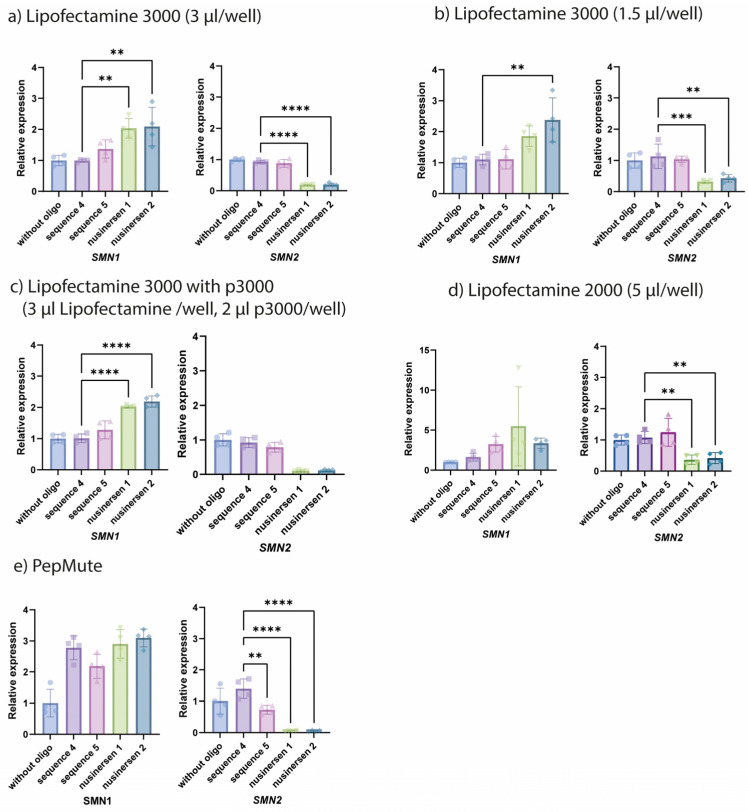
The effect of nusinersen (final concentration 200 nM) and control oligonucleotides on *SMN1* and *SMN2* expression in GM03813 cells (values normalized to control (no oligonucleotides added)). Oligonucleotides were tested at 200 nM concentration in GM03813 cells using five different lipofection reagents/protocols: Lipofectamine 3000 with 3 μL of Lipofectamine per well (**a**), Lipofectamine 3000 with 1.5 μL of Lipofectamine per well (**b**), Lipofectamine 3000 with the addition of p3000 (3 μL of Lipofectamine per well and 2 μL of p3000 per well) (**c**), Lipofectamine 2000 (5 μL of Lipofectamine per well) (**d**) and PepMute (**e**). ** *p*  <  0.01, *** *p*  <  0.001, **** *p*  <  0.0001. Two independent lipofection experiments were performed in duplicate. For qPCR, three technical replicates were performed. For statistical analysis, 1-way Anova was used for data with normal distribution and Kruskal–Wallis for data without normal distribution. Results were compared to sequence 4. Results are shown as mean ± SD.

The results of the *SMN1* and *SMN2* expression are shown in Figure 4. The results indicate that a higher concentration of Lipofectamine 3000 results in a more prominent effect of nusinersen on *SMN1* expression compared to a lower concentration of Lipofectamine 3000. When p3000 reagent was added to the Lipofectamine 3000, the effect was comparable to Lipofectamine 3000 without p3000 reagent and resulted in approximately 100% increase in *SMN1* expression. In contrast to Lipofectamine 3000, lipofection of nusinersen with Lipofectamine 2000 did not result in significant overexpression of *SMN1*. Moreover, the expression of *SMN1* was not comparable between the negative controls. When PepMute was used for transfection, no significant differences in *SMN1* expression were observed between negative controls, sequence 4, sequence 5 and both batches of nusinersen.

The results also show that the decrease in *SMN2* expression was more prominent when a higher concentration of Lipofectamine 3000 was used compared to a lower concentration (80% vs. 62–72% decrease). When the p3000 reagent was used, the expression of *SMN2* decreased by 88–89%. When Lipofectamine 2000 was used, the expression decreased by approximately 75%. In the presence of PepMute, the SMN2 expression decreased by 93%; however, the levels of SMN2 were not comparable between controls (e.g., sequence 4 vs. no oligonucleotides added or sequence 5).

The results indicate that Lipofectamine 3000 with a higher concentration of Lipofectamine reagent is the most efficient for achieving optimal results.

### 3.4. The Effect of Nusinersen on SMN Protein Expression in HEK293 and GM03813

For the evaluation of SMN protein expression, we first tested antibodies from four different manufacturers in cell lysates of HEK293, HEK293T, HepG2, and GM03813 cells and plasma samples as a negative control (Supplementary Figure S6a). When Abnova, Sigma and BD Biosciences antibodies were used, multiple bands were seen in lysates of HEK293 and HEK293T. Also, in GM03813 cell lysate, we noticed a strong band at approximately 38 kDa for SMN with all four antibodies tested. With all the antibodies tested, we also observed an additional band with higher molecular mass in HepG2 lysates. Plasma was negative for SMN protein when tested with antibodies from Abnova, BD Biosciences and Milipore, while Sigma antibody detected a protein with a lower molecular mass, which was also in this plasma sample.

Next, we evaluated the four antibodies on recombinant full-length SMN, SMNΔ7, and full-length SMN overexpression lysate (Supplementary Figure S6b). When we used antibodies from Abnova, we observed a signal for the SMN recombinant protein at about 38 kDa, while no band was seen for the SMNΔ7 control sample. There was no signal for SMN in the cell lysate sample. When we tested antibodies from Sigma, BD Biosciences and Millipore, we saw similar profiles of bands. A single band at around 38 kDa, which corresponds to SMN protein, was seen in the sample with recombinant SMN protein and also in the SMN overexpression cell lysate. There were four bands for recombinant SMNΔ7 protein; two at approximately 30 kDa, one at approximately 48 kDa and the strongest one at approximately 53 kDa, which most probably corresponds to the SMNΔ7-GST protein.

**Figure 5:**
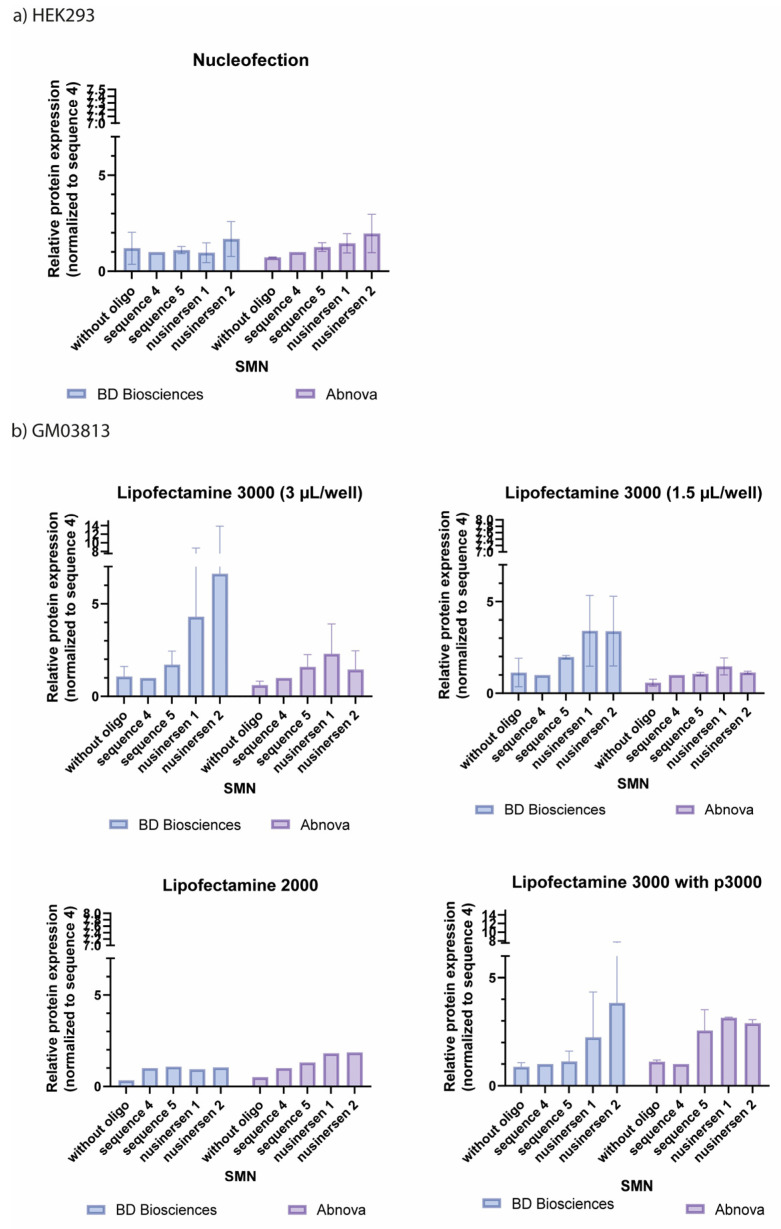
The effect of nusinersen on SMN protein expression. The effect of nusinersen was determined in HEK293 (**a**) and GM03813 (**b**). For HEK293 (highest seeding number), nucleofection was used. For GM03813, 4 different transfection reagents were used: Lipofectamine 3000 with 3 μL Lipofectamine 3000/well, Lipofectamine 3000 with 1.5 μL Lipofectamine 3000/well, Lipofectamine 3000 with p3000 and Lipofectamine 2000. SMN protein was detected using antibodies from BD Biosciences and Abnova. α-tubulin was used as a reference. The signal of the SMN protein was normalized to α-tubulin. Two independent experiments were performed, except for Lipofectamine 2000, where one experiment was performed.

We further examined the effects of nusinersen in HEK293 and GM03813 cells at the protein level, using specific and validated antibodies against full-length SMN, from BD Biosciences and Abnova (Figure 5, Supplementary Figures S7 and S8).

The results show that when Lipofectamine 3000 was used (3 and 1.5 μL/well as well as with p3000), the SMN overexpression was more than 2-fold higher, as compared to Lipofectamine 2000 (antibodies from BD Biosciences). When antibodies from BD Biosciences were used, the effect was the highest with Lipofectamine 3000 (3 μL/well) as compared to Lipofectamine 3000 (1.5 μL/well) or Lipofectamine 3000 with p3000. Also, when the Abnova antibody was used, the observed difference was higher with a higher amount of Lipofectamine 3000 (3 μL vs. 1.5 μL).

The results indicate that the effect of Nusinersen is also observed on a protein level with approximately a 2-fold increase. Similar to the results of mRNA expression, the best results are observed with higher HEK293 seeding concentrations and using Lipofectamine 3000 in GM03813.

### 3.5. The Effect of Nusinersen Dilution on SMN Gene and Protein Expression in HEK293 and GM03813

At last, we aimed to determine whether the effect of nusinersen is dose-dependent at the mRNA and protein levels.

**Figure 6:**
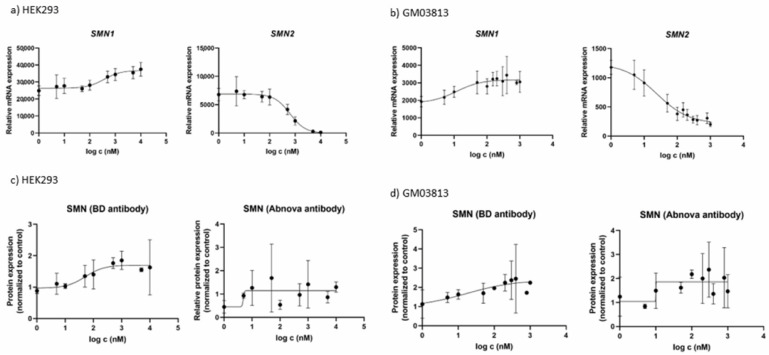
The effect of nusinersen dilution on *SMN* gene and SMN protein expression. Transcript expression was determined by qPCR in HEK293 (**a**) and GM03813 (**b**) cells, and protein expression was assessed by Western blot in HEK293 (**c**) and GM03813 (**d**) cells. At least two independent experiments were performed.

In HEK293 cells, increasing nusinersen concentrations resulted in a progressive increase in *SMN1* expression, reaching a plateau at 1 μM, while *SMN2* expression decreased with increasing nusinersen concentration. The calculated EC_50_ values in HEK293 cells were 293 nM for *SMN1* and 627 nM for *SMN2* (Figure 6a). Similarly, in GM03813 fibroblasts, *SMN1* expression increased with increasing nusinersen concentration and reached a plateau at 150 nM (Figure 6b), whereas *SMN2* expression decreased correspondingly. The EC_50_ in GM03813 cells was 10 nM for *SMN1* and 24 nM for *SMN2*.

Protein expression analysis by Western blot was performed to independently verify nusinersen-induced effects observed at the transcript level. Experiments were conducted using two independent biological replicates. Due to the semi-quantitative nature of Western blotting and its dependence on antibody performance, protein data were interpreted with appropriate caution and were not intended for precise quantitative comparisons.

Consistent with the transcript-level findings, SMN protein expression in both HEK293 and GM03813 cells increased in a dose-dependent manner with increasing nusinersen concentrations (Figure 6c,d, Supplementary Figure S9). Overall, the SMN-specific antibody from BD Biosciences generated more consistent results, whereas data obtained using the Abnova antibody showed greater variability.

## 4. Discussion

SMA is one of the most common genetic diseases in children, and if left untreated, it can often be fatal or severely impact the quality of life. The discovery of nusinersen has brought a major breakthrough in the treatment of SMA. At the same time, it opened a possibility for the development of new drugs for the treatment of SMA. In patients, oligonucleotide-based therapeutics enter the cell without the need for a delivery system. Although the mechanisms are poorly understood, it is known, mainly from in vitro experiments, that drugs enter the cells via a productive or non-productive way [25]. First, the oligonucleotide is taken up by clathrin-coated endocytotic vesicles. Afterward, the productive way mediated by several specific proteins (e.g., TCP1 complex and Hsp90) leads the drug to the target site. On the other hand, a non-productive pathway leads to drug sequestration without reaching the target molecule and inducing the desired effect. The mechanism is described in more detail by Khorkova et al. [25].

This work provides a systematically optimized and experimentally justified in vitro framework for evaluating *SMN2*-targeting antisense oligonucleotides. By systematically defining optimal experimental conditions in two widely used human cellular models, we demonstrate how parameters such as delivery strategy, cell density, and analytical tools influence apparent efficacy and potency. These findings are directly relevant for the comparative assessment and prioritization of novel antisense oligonucleotide therapeutics in early-stage development.

To implement this framework, we comprehensively evaluated various methods for nusinersen transfection. Given the continuous development of transfection technologies and the emergence of new reagents, systematic optimization remains necessary for reliable drug testing. Experiments in our research were performed with two complementary cell lines, HEK293 and GM03813. HEK293 is a human embryonic kidney cell line with epithelial morphology transformed with sheared human adenovirus type 5 DNA [26]. It has several advantages and is a suitable cell model for human hereditary disease research because it has high reproduction, is of human origin and is susceptible to various transfection methods [27]. It is particularly suitable for the study of SMA because it has high expression of *SMN1* and *SMN2*. The other cell line, GM03813, is a fibroblast-type isolated from a 3-year-old patient with spinal muscular atrophy type l [28,29], and therefore, provides a disease-relevant cellular context. Both cell lines have been widely used in published studies and patent literature investigating antisense oligonucleotides targeting *SMN2* [7,19,20,30,31,32]. Accordingly, HEK293 cells were used as a controlled mechanistic model to study delivery-dependent effects on *SMN2* splicing, whereas GM03813 fibroblasts served as a disease-relevant system to assess nusinersen activity in an endogenous SMA genetic background.

A limitation of the present study is the absence of direct quantitative measurement of oligonucleotide uptake. Instead, delivery efficiency was assessed through functional readouts reflecting productive intracellular delivery, which represents the biologically relevant determinant of antisense oligonucleotide activity.

For nusinersen transfection in HEK293 cells, nucleofection was employed, enabling direct delivery of nucleic acids into the cytoplasm and nucleus [33]. Although nucleofection has been reported to achieve high transfection efficiency in HEK293 cells (up to 93%), it is also associated with reduced cell survival (approximately 72%) [34]. The significant toxicity of nucleofection was also observed in our experiments, so a higher number of cell seeding is recommended. The levels of *SMN1* mRNA increased by approximately 40% at the highest nusinersen concentration, while SMN protein levels increased by 1.5–2.3-fold, which is consistent with findings by Hua et al., who reported a 2.1-fold increase in SMN protein expression [20]. Hua et al. [20] performed the experiments in a similar manner with HEK293 and GM03813. However, in HEK293, the cells were transfected with SMN1 and SMN2 minigenes.

In GM03813 cells, lipofection was preferred over electroporation, in line with previous studies demonstrating improved efficiency and tolerability [20]. We evaluated three commercially available reagents: Lipofectamine 3000 and Lipofectamine 2000, which are lipid-based cationic reagents that interact with the negatively charged phosphate scaffold of nucleic acids and facilitate their entrapment and enter the cell by endocytosis [33], and PepMute, which is based on virus-derived peptide and serves as so called cell-penetrating peptide [35]. Our results show that a higher concentration (3 μL/well) of Lipofectamine 3000 yields optimal results, and the p3000 reagent did not bring any additional effect. Lipofectamine 2000, although used in previous studies [36,37,38], caused substantial cytotoxicity in GM03813 cells, consistent with reports in other cell types [39]. *SMN1* expression increased by 2–2.2-fold when nusinersen was delivered using Lipofectamine 3000. The results are similar to those of Hua et al., who tested nusinersen in the GM03813 cell line at a concentration of 100 nM using lipofectin, with a 1.8-fold increase in full-length SMN transcript levels (determined by RT-PCR with radioactive detection) [20].

For SMN protein detection, four commercially available anti-SMN antibodies were initially evaluated. Using antibodies from Abnova, we observed a signal for recombinant full-length SMN protein at approximately 38 kDa, whereas no band was seen in the SMNΔ7 control sample, suggesting that these Abnova polyclonal antibodies are specific for full-length SMN. Western blot experiments performed with other antibodies showed that, unlike Abnova’s antibodies, the antibodies from Sigma, BD Biosciences, and Millipore are not specific for full-length SMN but recognize both full-length SMN and the truncated protein. These conclusions are supported by the manufacturer Abnova, as antibodies bind to the C-terminus of the SMN protein, which is the only difference between full-length SMN and SMNΔ7, because SMNΔ7 is shorter at the C-terminus. In addition, BD Biosciences provides information about the immunogen, a polypeptide comprising 14–174 amino acids of the SMN protein, and this sequence is identical in SMN and SMNΔ7 proteins. Therefore, for further experiments, we selected SMN-specific polyclonal antibodies from Abnova and monoclonal antibodies from BD Biosciences with a known target sequence, which is necessary information for the correct description and validation of antibodies [40]. This antibody was also used in patents and published papers [7,19,20]. When both antibodies were used for SMN detection at different nusinersen concentrations, we observed that the results obtained with the BD Biosciences antibodies were scattered but more consistent compared with those obtained with Abnova antibodies. This suggests that the BD Biosciences antibodies are more suitable for analyses of SMN expression, although these antibodies do not distinguish between full-length SMN and SMNΔ7 but recognize both forms. Because of the constant evolution of antibodies, antibodies used in published reports may no longer be available. Scientists working in this field have to carefully select and validate new antibodies for each study [40]. These data thus highlight the important issue of antibody quality, which is one of the main reasons for the reproducibility crisis in bioscience [41,42,43].

Protein-level analysis was included to verify that nusinersen-induced transcript changes translate into corresponding alterations in SMN protein expression. Although the number of biological replicates was limited and Western blotting is inherently semi-quantitative, protein expression patterns were consistent across experiments and aligned with transcript-level data. These results confirm the biological effect of nusinersen while acknowledging that the experimental design does not permit rigorous statistical evaluation of subtle differences or precise potency estimation at the protein level.

## 5. Conclusions

In this study, we systematically optimized and benchmarked in vitro experimental conditions for evaluating the activity of the antisense oligonucleotide nusinersen in two widely used human cellular models. We show that nucleofection of HEK293 cells is associated with substantial cytotoxicity, necessitating higher cell-seeding densities to achieve robust and reproducible readouts. In GM03813 patient-derived fibroblasts, Lipofectamine 3000 at higher reagent concentrations provided the most efficient and best-tolerated delivery conditions. Under optimized conditions, nusinersen exhibited clear dose-dependent effects at both the transcript and protein levels.

Importantly, this work defines a systematically optimized and reproducible in vitro framework for assessing *SMN2*-targeting antisense oligonucleotides, demonstrating how experimental parameters critically influence apparent efficacy and potency. These findings provide practical guidance for comparative evaluation and prioritization of antisense oligonucleotide candidates and can be readily applied to the development and screening of existing and novel therapeutics for SMA and other splicing-related genetic diseases.

## Figures and Tables

**Table 1:** Nusinersen sample information.

Nusinersen Sample Name	Nusinersen Sources	Lot
Nusinersen 1	Spinraza (Biogen Netherlands B.V., Badhoevedorp, The Netherlands)	KS 1426169
Nusinersen 2	Spinraza (Biogen Netherlands B.V., Badhoevedorp, The Netherlands)	KS 1425927

**Table 2:** Negative control oligonucleotides.

Name	Sequence	Reference
Sequence 4	5′-TTGTATTCTATGTTT-3′	[19,20]
Sequence 5	5′-TGCATCTCATTGTAG-3′	[7]

**Table 3:** Assays for SMN1 and SMN2.

Gene/Primer	Sequence	Manuf.	Batch	Gene Region
*SMN* forwards	5′-GCTCACATTCCTTAAATTAAGGAGAAA-3′	Sigma	ST04908260	exon 7–exon 8
*SMN* reverse	5′-TCCAGATCTGTCTGATCGTTTCTT-3′	Sigma	ST04908261	exon 8
Without Exon 7 forward	5′-TGGCTATCATACTGGCTATTATATGGAA-3′	Sigma	ST04908262	exon 6–exon 8
*SMN* probe [21]	5′-CTGGCATAGAGCAGCACTAAATGACACCAC-3′	Thermo Fisher	/	exon 8

**Table 4:** List of reference genes.

Gene	Manufacturer	Assay	Lot
*POLR2A*	Thermo Fisher Scientific	Hs00172187_m1	1921262
*HPRT1*	Thermo Fisher Scientific	Hs99999909_m1	1907007
*GAPDH*	Thermo Fisher Scientific	Hs02758991_g1	1865044

**Table 5:** Antibodies for Western blot optimization.

Target	Manufacturer	Catalog Number	Lot	Final Dilution/Concentration
SMN	Abnova	PAB12734	804-577	1:500
SMN	Sigma	SAB5300203	1106211	1:1000
SMN	BD Biosciences (San Jose, CA, USA)	610646	9338655	1:1000
SMNΔ7	Milipore	MABE230	3420862	0.5 μg/mL

## Data Availability

The original contributions presented in this study are included in the article/Supplementary Materials. Further inquiries can be directed to the corresponding authors.

## Supplementary Materials

The following supporting information can be downloaded at: https://www.mdpi.com/article/10.3390/pharmaceutics18060652/s1, Figure S1: The effect of electric pulse on HEK293—no oligonucleotide added. Figure S2: Nucleofection of HEK293 with nusinersen 1. Figure S3: The effect of transfection reagents on GM03813. The figure shows GM03813 cells after adding transfection reagents. First time point for obtaining figures was medium change (6 h for all lipofection protocols, except for PepMute, which was 24 h). The photos were taken every 24 h. The photos at the last time point (72 h) were taken before cell harvesting for RNA or protein isolation. Figure S4: Transfection of GM03813 with nusinersen. The figure shows GM03813 cells after transfection with nusinersen. First time point for obtaining figures was a change in medium (6 h for all transfection protocols, except for PepMute, which was 24 h). The photos were taken every 24 h. The photos at the last time point (72 h) were taken before cell harvesting for RNA and protein isolation. Figure S5: Lipofection of GM03813 with sequence 4. The figure shows GM03813 after lipofection of sequence 4 using the reagent Lipofectamine 2000. The photo was taken 72 h after the addition of the liposome with oligonucleotide. Figure S6: Anti-SMN antibody validation. Validation included: (a) Different cell lines (HEK293, HEK293T, GM03813, HepG2) and plasma as a negative control, (b) SMN and SMNΔ7 recombinant proteins as well as SMN overexpression lysate. Antibodies from four different manufacturers were tested: Abnova, Sigma, BD Biosciences and Millipore. The catalog numbers and additional information are shown in Table 3. Figure S7: The effect of nusinersen on SMN protein expression in HEK293. Three different seeding concentrations were used: 2 × 10^5^ (a), 5 × 10^5^ (b), 1 × 10^6^ (b) and two different anti-SMN antibodies, from BD Biosciences and Abnova. Alpha-tubulin was used as a reference. Figure S8: The effect of nusinersen in GM03813. Lipofectamine 3000 with 3 μL Lipofectamine 3000/well (a,b), Lipofectamine 3000 with 1.5 μL Lipofectamine 3000/well (a,b), Lipofectamine 3000 with p3000 (c,d) and Lipofectamine 2000 (c,d). SMN protein was detected using antibodies from BD Biosciences (a,c) and Abnova (b,d). Alpha-tubulin was used as a reference. Figure S9: The effect of nusinersen dilution curve on SMN protein expression in HEK293 (a) and GM03813 (b). In HEK293, concentrations from 1 nM to 10 μM were tested (a). In GM03813, concentrations from 1 nM to 1000 nM were tested (b). Alpha-tubulin was used as a reference. Table S1: Complex preparation for Lipofectamine 3000; Table S2: Complex preparation for Lipofectamine 3000 with p3000 reagent; Table S3: Complex preparation for Lipofectamine 2000; Table S4: Complex preparation for PepMute.

## Abbreviations

The following abbreviations are used in this manuscript:

SMA	Spinal muscular atrophy
SMN	Survival motor neuron
HEK	Human embryonic kidney
ANOVA	Analysis of variance

## References

[1] Ross L.F., Kwon J.M. (2019). Spinal Muscular Atrophy: Past, Present, and Future. Neoreviews.

[2] Arnold E.S., Fischbeck K.H. (2018). Spinal muscular atrophy. Handbook of Clinical Neurology.

[3] Bennett C.F. (2019). Therapeutic Antisense Oligonucleotides are Coming of Age. Annu. Rev. Med..

[4] Arnold W.D., Kassar D., Kissel J.T. (2015). Spinal muscular atrophy: Diagnosis and management in a new therapeutic era. Muscle Nerve.

[5] Chiriboga C.A. (2017). Nusinersen for the treatment of spinal muscular atrophy. Expert Rev. Neurother..

[6] Lorson C.L., Hahnen E., Androphy E.J., Wirth B. (1999). A single nucleotide in the SMN gene regulates splicing and is responsible for spinal muscular atrophy. Proc. Natl. Acad. Sci. USA.

[7] Hua Y., Vickers T.A., Okunola H.L., Bennett C.F., Krainer A.R. (2008). Antisense masking of an hnRNP A1/A2 intronic splicing silencer corrects SMN2 splicing in transgenic mice. Am. J. Hum. Genet..

[8] Lim S.R., Hertel K.J. (2001). Modulation of survival motor neuron pre-mRNA splicing by inhibition of alternative 3′ splice site pairing. J. Biol. Chem..

[9] Monani U.R., Lorson C.L., Parsons D.W., Prior T.W., Androphy E.J., Burghes A.H., McPherson J.D. (1999). A single nucleotide difference that alters splicing patterns distinguishes the SMA gene SMN1 from the copy gene SMN2. Hum. Mol. Genet..

[10] Singh R.N., Singh N.N. (2018). Mechanism of Splicing Regulation of Spinal Muscular Atrophy Genes. Advances in Neurobiology.

[11] Thery C., Witwer K.W., Aikawa E., Alcaraz M.J., Anderson J.D., Andriantsitohaina R., Antoniou A., Arab T., Archer F., Atkin-Smith G.K. (2018). Minimal information for studies of extracellular vesicles 2018 (MISEV2018): A position statement of the International Society for Extracellular Vesicles and update of the MISEV2014 guidelines. J. Extracell. Vesicles.

[12] Claborn M.K., Stevens D.L., Walker C.K., Gildon B.L. (2019). Nusinersen: A Treatment for Spinal Muscular Atrophy. Ann. Pharmacother..

[13] Maggi L., Bello L., Bonanno S., Govoni A., Caponnetto C., Passamano L., Grandis M., Trojsi F., Cerri F., Ferraro M. (2020). Nusinersen safety and effects on motor function in adult spinal muscular atrophy type 2 and 3. J. Neurol. Neurosurg. Psychiatry.

[14] De Vivo D.C., Bertini E., Swoboda K.J., Hwu W.L., Crawford T.O., Finkel R.S., Kirschner J., Kuntz N.L., Parsons J.A., Ryan M.M. (2019). Nusinersen initiated in infants during the presymptomatic stage of spinal muscular atrophy: Interim efficacy and safety results from the Phase 2 NURTURE study. Neuromuscul. Disord..

[15] Singh N.K., Singh N.N., Androphy E.J., Singh R.N. (2006). Splicing of a critical exon of human Survival Motor Neuron is regulated by a unique silencer element located in the last intron. Mol. Cell. Biol..

[16] Roberts T.C., Langer R., Wood M.J.A. (2020). Advances in oligonucleotide drug delivery. Nat. Rev. Drug Discov..

[17] Scudiero D.A., Polinsky R.J., Brumback R.A., Tarone R.E., Nee L.E., Robbins J.H. (1986). Alzheimer disease fibroblasts are hypersensitive to the lethal effects of a DNA-damaging chemical. Mutat. Res..

[18] Stabley D.L., Holbrook J., Harris A.W., Swoboda K.J., Crawford T.O., Sol-Church K., Butchbach M.E.R. (2017). Establishing a reference dataset for the authentication of spinal muscular atrophy cell lines using STR profiling and digital PCR. Neuromuscul. Disord..

[19] Brenda F., Baker A.R.K., Hua Y. (2013). Compositions and Methods for Modulation of SMN2 Splicing. U.S. Patent.

[20] Hua Y., Vickers T.A., Baker B.F., Bennett C.F., Krainer A.R. (2007). Enhancement of SMN2 exon 7 inclusion by antisense oligonucleotides targeting the exon. PLoS Biol..

[21] Naryshkin N.A., Weetall M., Dakka A., Narasimhan J., Zhao X., Feng Z., Ling K.K., Karp G.M., Qi H., Woll M.G. (2014). Motor neuron disease. SMN2 splicing modifiers improve motor function and longevity in mice with spinal muscular atrophy. Science.

[22] Dastpeyman M., Sharifi R., Amin A., Karas J.A., Cuic B., Pan Y., Nicolazzo J.A., Turner B.J., Shabanpoor F. (2021). Endosomal escape cell-penetrating peptides significantly enhance pharmacological effectiveness and CNS activity of systemically administered antisense oligonucleotides. Int. J. Pharm..

[23] Farrelly-Rosch A., Lau C.L., Patil N., Turner B.J., Shabanpoor F. (2017). Combination of valproic acid and morpholino splice-switching oligonucleotide produces improved outcomes in spinal muscular atrophy patient-derived fibroblasts. Neurochem. Int..

[24] Bustin S.A., Benes V., Garson J.A., Hellemans J., Huggett J., Kubista M., Mueller R., Nolan T., Pfaffl M.W., Shipley G.L. (2009). The MIQE guidelines: Minimum information for publication of quantitative real-time PCR experiments. Clin. Chem..

[25] Khorkova O., Wahlestedt C. (2017). Oligonucleotide therapies for disorders of the nervous system. Nat. Biotechnol..

[26] Cell Culture Select Tool. https://www.thermofisher.com/si/en/home/technical-resources/cell-lines/2/cell-lines-detail-176.html.

[27] Abaandou L., Quan D., Shiloach J. (2021). Affecting HEK293 Cell Growth and Production Performance by Modifying the Expression of Specific Genes. Cells.

[28] GM03813. https://www.coriell.org/0/sections/Search/Sample_Detail.aspx?Ref=GM03813&product=CC.

[29] Coovert D.D., Le T.T., McAndrew P.E., Strasswimmer J., Crawford T.O., Mendell J.R., Coulson S.E., Androphy E.J., Prior T.W., Burghes A.H. (1997). The survival motor neuron protein in spinal muscular atrophy. Hum. Mol. Genet..

[30] Brenda F., Baker A.R.K., Hua Y. (2023). Compositions and Methods for Modulation of SMN2 Splicing. U.S. Patent.

[31] Brenda F., Baker A.R.K., Hua Y. (2020). Compositions and Methods for Modulation of SMN2 Splicing. U.S. Patent.

[32] Brenda F., Baker A.R.K., Hua Y. (2019). Compositions and Methods for Modulation of SMN2 Splicing. U.S. Patent.

[33] Electroporation and Nucleofector^®^ Technology. https://bioscience.lonza.com/lonza_bs/SI/en/nucleofector-technology.

[34] Maurisse R., De Semir D., Emamekhoo H., Bedayat B., Abdolmohammadi A., Parsi H., Gruenert D.C. (2010). Comparative transfection of DNA into primary and transformed mammalian cells from different lineages. BMC Biotechnol..

[35] PepMute™ siRNA Transfection Reagent. https://signagen.com/products/pepmute-sirna-transfection-reagent/.

[36] Singh N.N., Shishimorova M., Cao L.C., Gangwani L., Singh R.N. (2009). A short antisense oligonucleotide masking a unique intronic motif prevents skipping of a critical exon in spinal muscular atrophy. RNA Biol..

[37] Singh N.N., Luo D., Singh R.N. (2018). Pre-mRNA Splicing Modulation by Antisense Oligonucleotides. Methods Mol. Biol..

[38] Dickson A., Osman E., Lorson C.L. (2008). A negatively acting bifunctional RNA increases survival motor neuron both in vitro and in vivo. Hum. Gene Ther..

[39] Wang T., Larcher L.M., Ma L., Veedu R.N. (2018). Systematic Screening of Commonly Used Commercial Transfection Reagents towards Efficient Transfection of Single-Stranded Oligonucleotides. Molecules.

[40] Rizner T.L., Sasano H., Choi M.H., Odermatt A., Adamski J. (2016). Recommendations for description and validation of antibodies for research use. J. Steroid Biochem. Mol. Biol..

[41] Reiss P.D., Min D., Leung M.Y. (2014). Working towards a consensus for antibody validation. F1000Res.

[42] Pillai-Kastoori L., Heaton S., Shiflett S.D., Roberts A.C., Solache A., Schutz-Geschwender A.R. (2020). Antibody validation for Western blot: By the user, for the user. J. Biol. Chem..

[43] Pillai-Kastoori L., Schutz-Geschwender A.R., Harford J.A. (2020). A systematic approach to quantitative Western blot analysis. Anal. Biochem..

