# Supplementary material for: Optimizing In Vitro Efficacy Assessment of the Antisense Oligonucleotide Nusinersen in Human Cellular Models
Source: Pharmaceutics. 2026 May 26;18(6):652. doi: 10.3390/pharmaceutics18060652 (PMC13305313; doi:10.3390/pharmaceutics18060652)
Supplement: Supplementary file 1 [file pharmaceutics-18-00652-s001.zip › Supplementary Tables S1-S4.pdf]

Supplementary table S1: Complex preparation for Lipofectamine 3000.

Step	Reagent	Volume per one reaction (µL)
1. Lipofectamine 3000 reagent dilution	Opti-MEM	50
	Lipofectamine 3000 reagent	3 or 1.5
2. Oligonucleotide dilution	Opti-MEM	50
	Oligonucleotide	2
3. Add diluted oligo to Lipofectamine solution	Lipofectamine dilution	50
	Oligonucleotide dilution	50

Supplementary table S2: Complex preparation for Lipofectamine 3000 with p3000 reagent.

Step	Reagent	Volume per one reaction (µL)
1. Lipofectamine 3000 reagent dilution	Opti-MEM	50
	Lipofectamine 3000 reagent	3
2. Oligonucleotide dilution	Opti-MEM	50
	Oligonucleotide	2
	p3000 reagent	2
3. Add diluted oligo to Lipofectamine solution	Lipofectamine dilution	50
	Oligonucleotide dilution	50

Supplementary table S3: Complex preparation for Lipofectamine 2000.

Step	Reagent	Volume per one reaction (µL)
1. Lipofectamine 3000 reagent dilution	Opti-MEM	50
	Lipofectamine 2000 reagent	5
2. Oligonucleotide dilution	Opti-MEM	50
	Oligonucleotide	2
3. Add diluted oligo to Lipofectamine solution	Lipofectamine dilution	50
	Oligonucleotide dilution	50

Supplementary table S4: Complex preparation for PepMute.

Reagent	Volume per one reaction (μL)
PepMute buffer (working solution (1x))	75
Oligonucleotide	2
PepMute reagent	2
