# Supplementary figures and images for: Optimizing In Vitro Efficacy Assessment of the Antisense Oligonucleotide Nusinersen in Human Cellular Models
Source: Pharmaceutics. 2026 May 26;18(6):652. doi: 10.3390/pharmaceutics18060652 (PMC13305313; doi:10.3390/pharmaceutics18060652)

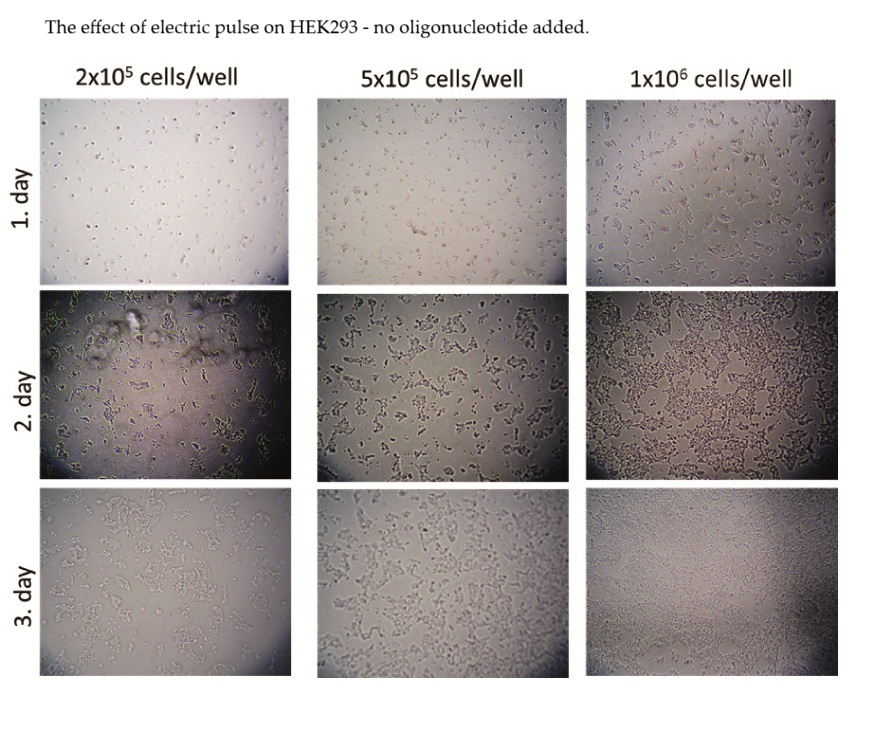

Supplement: Supplementary file 1 [file pharmaceutics-18-00652-s001.zip › Supplementary Figure S1.jpg]

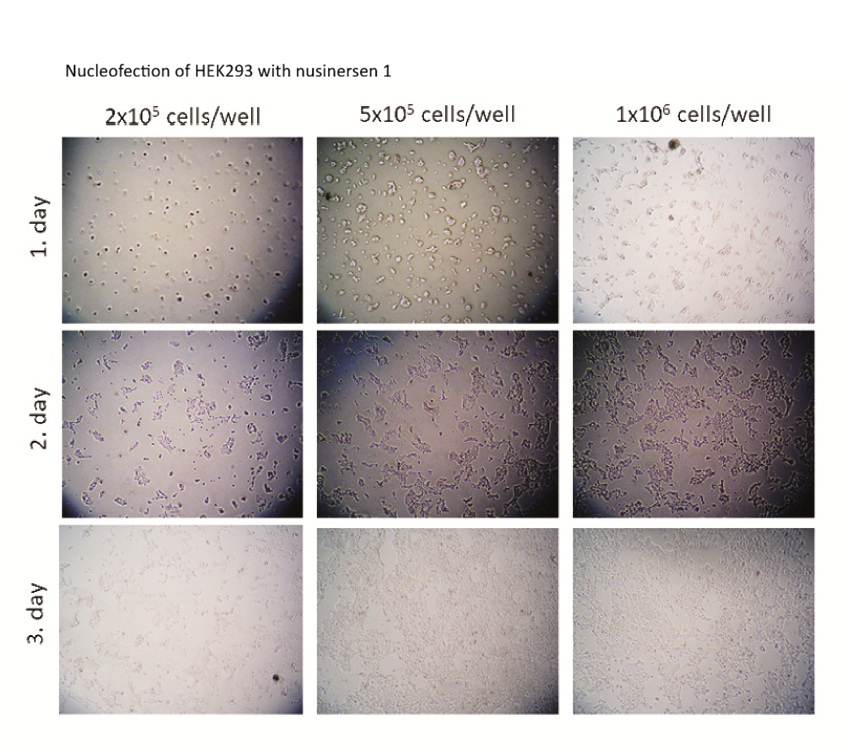

Supplement: Supplementary file 1 [file pharmaceutics-18-00652-s001.zip › Supplementary Figure S2.jpg]

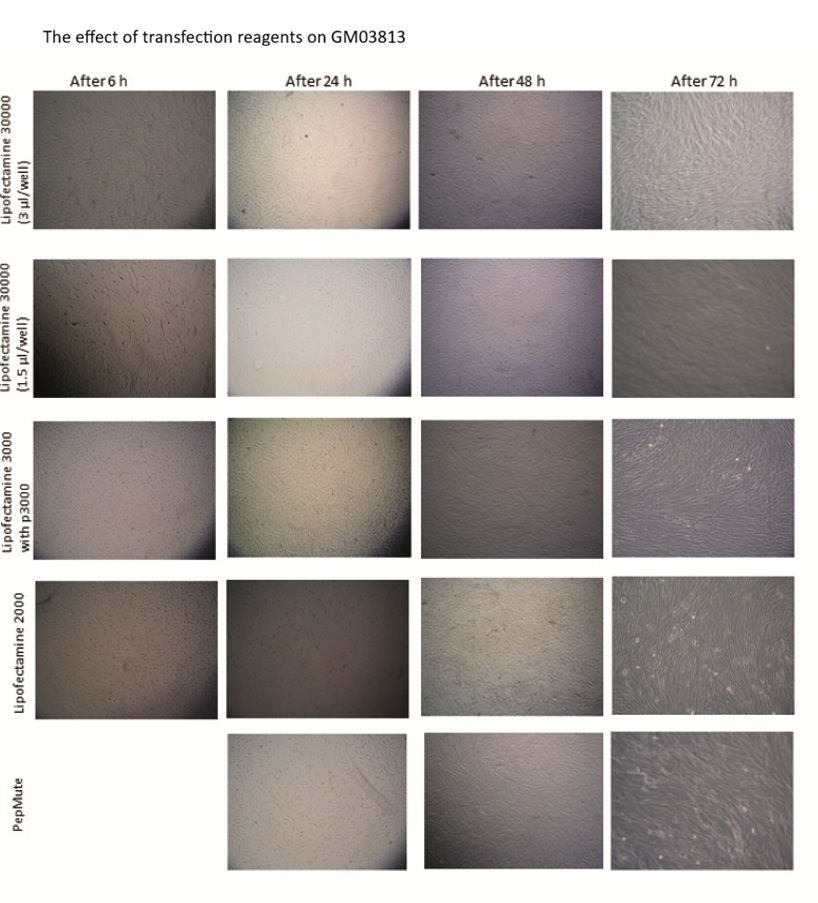

Supplement: Supplementary file 1 [file pharmaceutics-18-00652-s001.zip › Supplementary Figure S3.jpg]

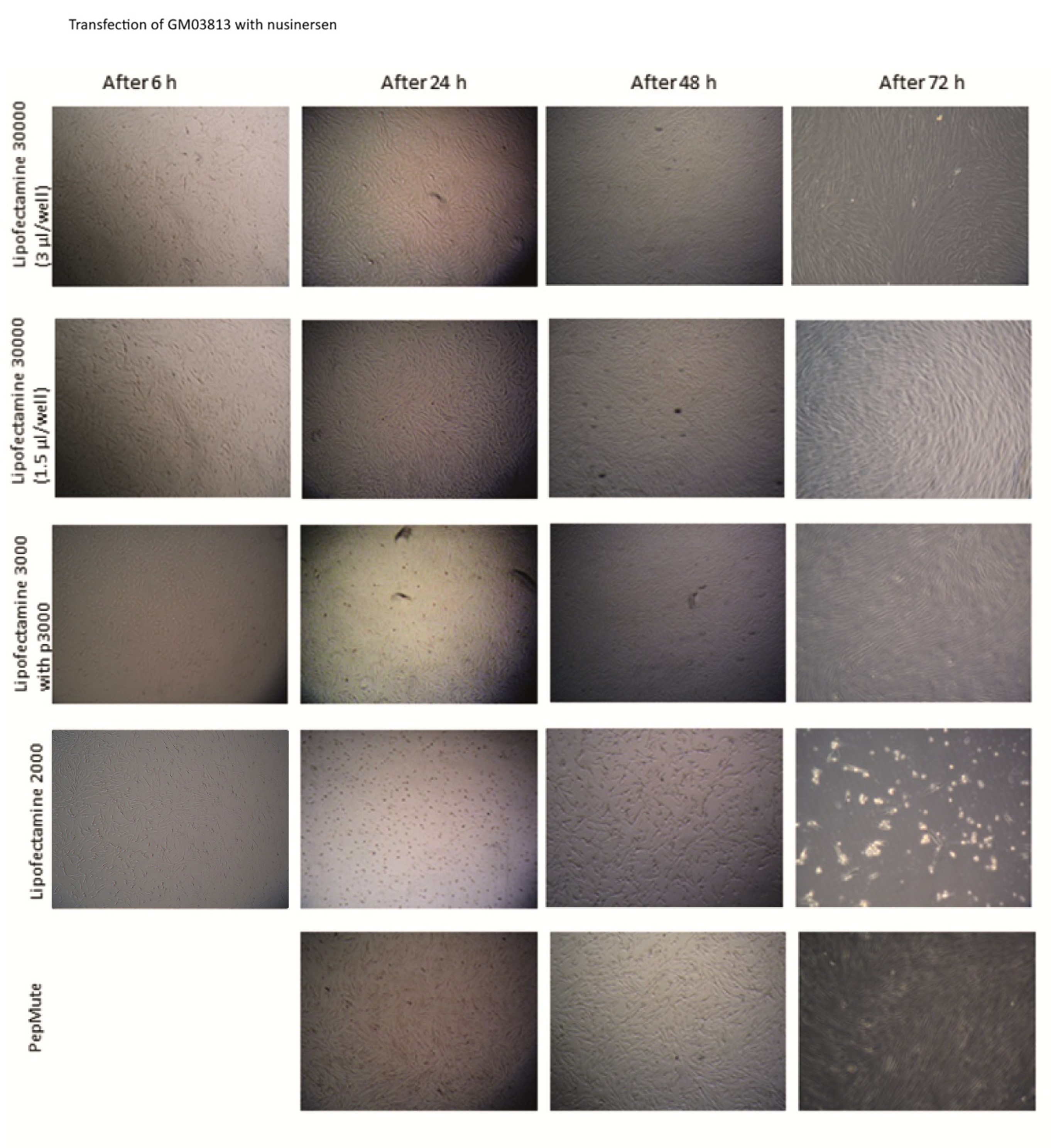

Supplement: Supplementary file 1 [file pharmaceutics-18-00652-s001.zip › Supplementary Figure S4.jpg]

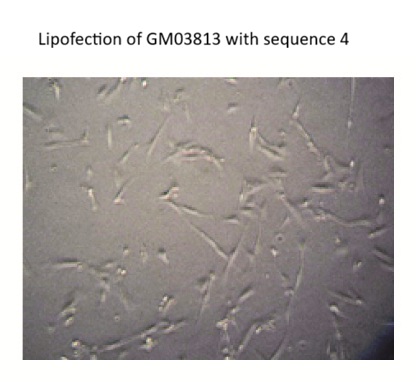

Supplement: Supplementary file 1 [file pharmaceutics-18-00652-s001.zip › Supplementary Figure S5.jpg]

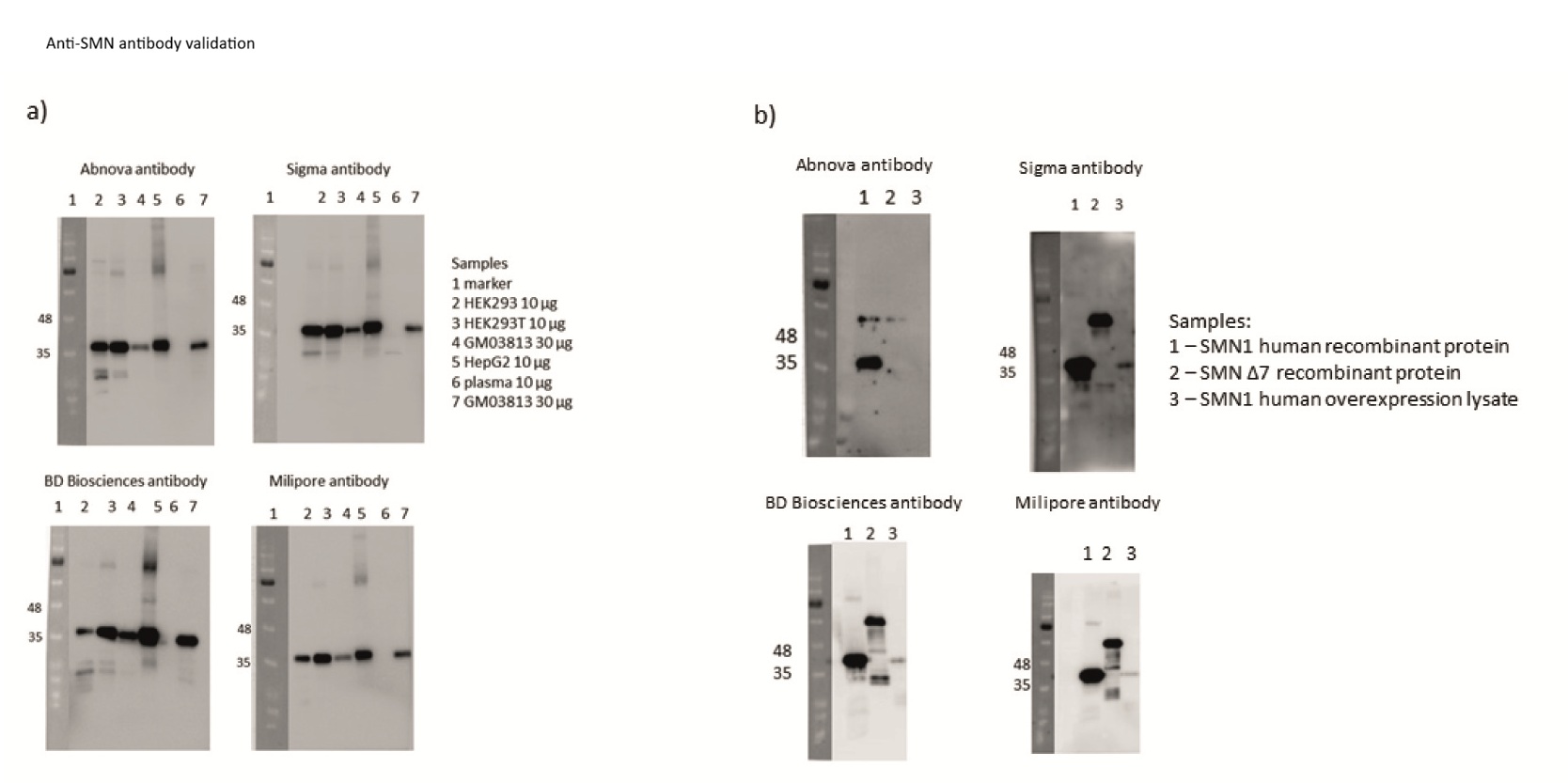

Supplement: Supplementary file 1 [file pharmaceutics-18-00652-s001.zip › Supplementary Figure S6.jpg]

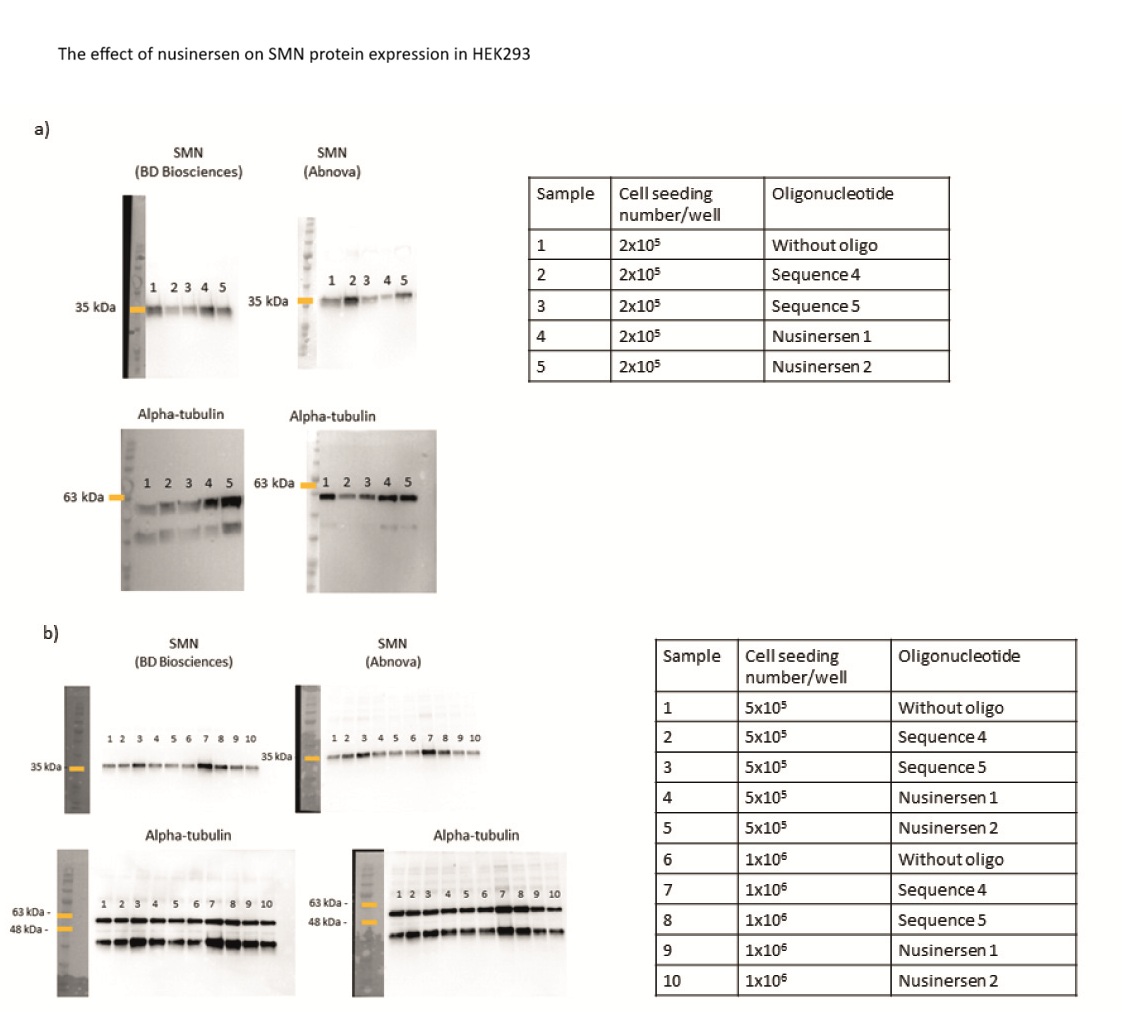

Supplement: Supplementary file 1 [file pharmaceutics-18-00652-s001.zip › Supplementary Figure S7.jpg]

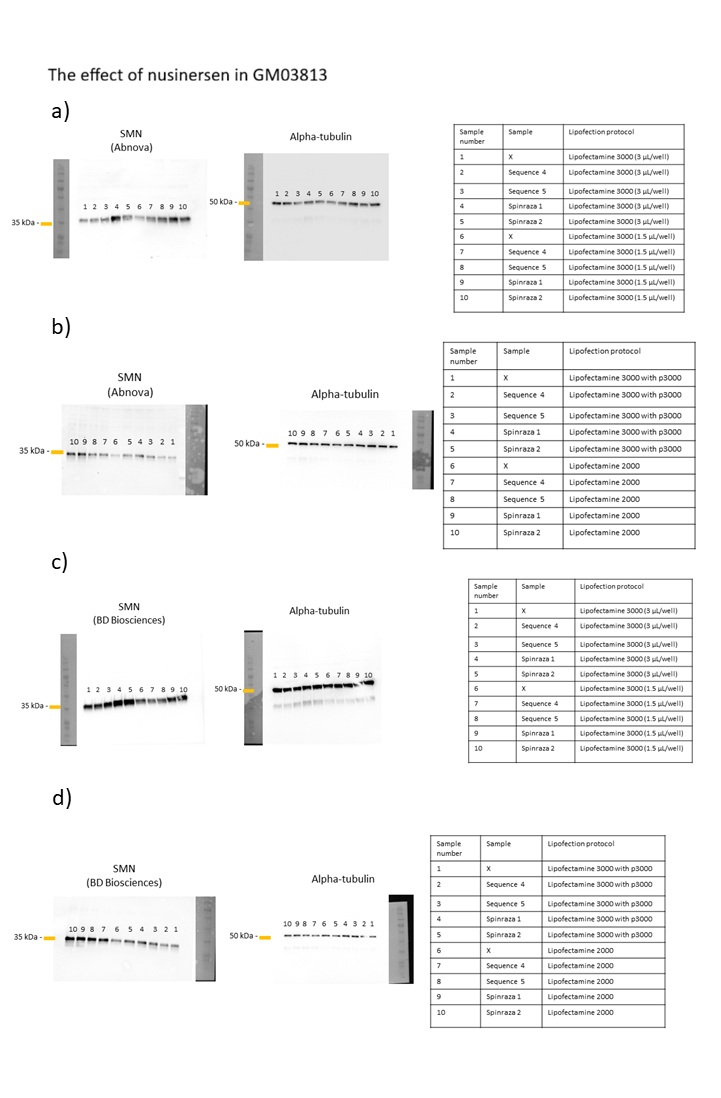

Supplement: Supplementary file 1 [file pharmaceutics-18-00652-s001.zip › Supplementary Figure S8.jpg]

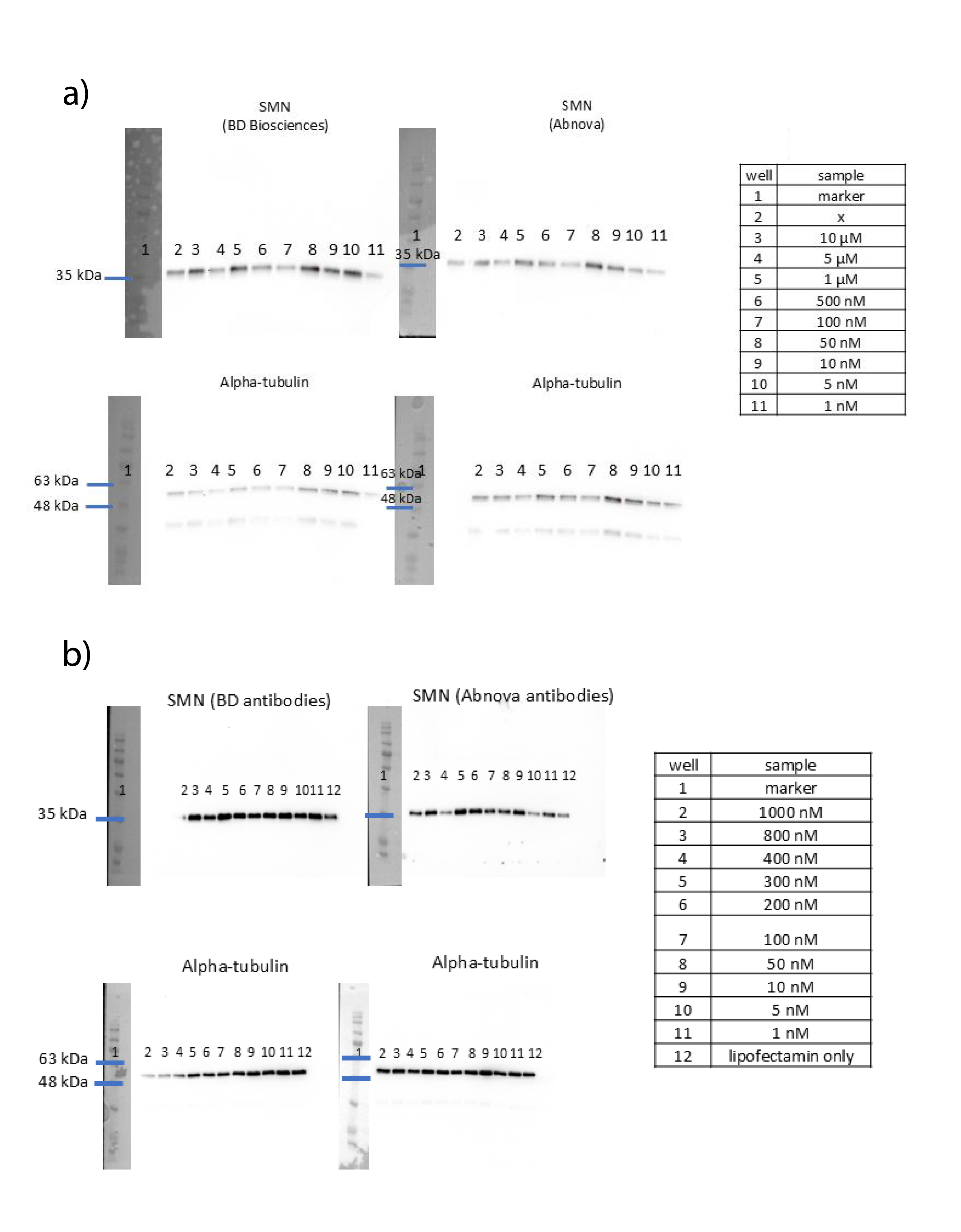

Supplement: Supplementary file 1 [file pharmaceutics-18-00652-s001.zip › Supplementary Figure S9.png]
